# Enzymatic decolorization of melanin by lignin peroxidase from *Phanerochaete chrysosporium*

**DOI:** 10.1038/s41598-020-76376-9

**Published:** 2020-11-19

**Authors:** Beenish Sadaqat, Nazia Khatoon, Aneela Younas Malik, Asif Jamal, Uzma Farooq, Muhammad Ishtiaq Ali, Huan He, Fang-Jing Liu, Hongguang Guo, Michael Urynowicz, Qiurong Wang, Zaixing Huang

**Affiliations:** 1 Department of Microbiology, Quaid-I-Azam University, Islamabad, 45320 Pakistan; 2 Instiute of Space Technology, Islamabad, 44000 Pakistan; 3 Key Laboratory of Coal Processing and Efficient Utilization of Ministry of Education, School of Chemical Engineering and Technology, China University of Mining and Technology, Xuzhou, 221116 China; 4 College of Safety and Emergency Management and Engineering, Taiyuan University of Technology, Taiyuan, 030024 China; 5 Department of Civil and Architectural Engineering, University of Wyoming, Laramie, 82071 USA; 6 Department of Animal Science, University of Wyoming, Laramie, WY 82071 USA

**Keywords:** Environmental microbiology, Applied microbiology, Fungi

## Abstract

Skin darkening results as a consequence of the accumulation of skin pigment melanin. To combat this, the amplitude of skin lightening agents are commercially available, most of which inhibit melanin synthesis. Decolorization of melanin is an alternative method of skin lightening. In this study, we show that lignin peroxidase (LiP), an extracellular enzyme purified from *Phanerochaete chrysosporium* NK-1 isolated from a forest soil can effectively degrade and decolorize melanin in vitro. Decolorization conditions including pH, temperature, incubation time, enzyme concentration, and mediator addition were investigated to optimize the reaction conditions. The results indicate that pH 3, 40 °C, 15 IU/ml, and 10 h incubation were the optimal conditions for the decolorization of the melanin. The use of the mediator, veratryl alcohol was also found effective to enhance the efficacy of the melanin decolonization, with up to 92% decolorization. The scanning electron microscopy results showed void spaces on the treated melanin granules as compared to the untreated sample, indicating the degradation of melanin. Changes in the fingerprint region of the melanin were observed. Between wavenumbers 1500–500 cm^−1^, for example, the presence of new peaks in the treated melanin at 1513, 1464, and 1139 cm^−1^ CH_2_, CH_3_ bend and C–O–C stretch represented structural changes. A new peak at 2144 cm^−1^ (alkynyl C≡C stretch) was also detected in the decolorized melanin. The cytotoxicity study has shown that the treated melanin and LiP have low cytotoxic effects; however, the mediator of veratryl alcohol could result in high mortality which suggests that its use should be meticulously tested in formulating health and skincare products. The findings of the study suggest that LiP produced by *Phanerochaete chrysosporium* has the potential to be used in the medical and cosmetic industries, particularly for the development of biobased cosmetic whitening agents.

## Introduction

Melanin is a group of complex, recalcitrant, and pervasive polycyclic bio-polymeric skin pigments produced by specialized skin cells called melanocytes ^1,2^. Its primary function is to protect skin against harmful UV radiations of sunlight by forming supranuclear caps around the DNA of the skin cells ^3^. Besides, it is responsible for different skin colors by absorbing various free radicals within the cytoplasm. Most of these skin lightening agents obtained from natural resources inhibit melanin production in the skin cells by inhibition of tyrosinase enzymes, suppression of pigment biosynthesis and through some alternative pathways. Various compounds like hydroquinone, mono-benzyl ethers of hydroquinone, mercury, corticosteroids, and arbutin are being used in the commercial formulation for skin whitening cosmetics, however, their applications have been associated with serious side effects. For example, mercury is reported to cause damage to kidney and lead has been reported to cause anxiety, depression, and psychosis ^4,5^. The use of corticosteroids may cause Cushing’s syndrome, diabetes, hypertension, adrenal insufficiency, immunosuppression, skin thinning, acne, dermatitis and hypertrichosis ^6^. Tain et al. (2009) reported that the use of arbutin in the skin whitening cosmetics was effective for concentrations up to 3% but further increase in the concentration caused cytotoxic effects. Considering these limitations, applications of these inhibitors in the skin whitening cosmetics has been broadly criticized by the health and safety authorities. It has been proposed that skin whitening cosmetics could reach up to US$ 155.44 billion by the year 2021 with an annual growth rate of 4.9%. Owing to the increasing demand for safe and natural skin whitening agents worldwide, enzymatic melanin decolonization has received considerable interest ^7–9^.

Microbial extracellular enzymes such as laccase, manganese peroxidase and lignin peroxidase (LiP) have been previously tested for melanin decolorization and considered as a potential eco-friendly alternative to the toxic chemicals ^1^. Among these, LiP was found very effective for the decolorization of melanin because of its high redox potential to oxidize veratryl alcohol (VA) than the other related enzymes. Despite high oxidation potential and efficient catalysis of melanin, volumetric production and difficulties in purification are the major limitations for commercial applications of LiP in skin whitening agents. In addition, LiP loses its enzymatic activity for melanin decolonization under excess hydrogen peroxide, which is considered critical for the oxidation of melanin. Besides its important role, H_2_O_2_ inactivates LiP thus reduce the catalytic performance of the enzyme.

The white-rot fungus, *Phanerochaete chrysosporium* has been reported as an efficient biological resource for the production of LiP. However, currently, the major obstacles in the application of this enzyme include the development of cost-effective fermentation processes, isolation of productive stains and purification of the enzyme since the fungus produces a mixture of 17 isoenzymes. In order to address low productivity and enzyme sensitivity issues, the present study was conducted to develop a cost-effective and efficient process for LiP production from an indigenous isolate of *Phanerochaete chrysosporium*. The fungus produces a comparatively high concentration of LiP with better catalytic efficiency at relatively lower enzyme concentrations than previously reported. The production was carried out in submerged fermentation using VA as the mediator. The optimum reaction conditions for melanin decolonization were investigated with purified LiP along with the determination of cytotoxicity effects of the purified enzyme.

## Results

### Decolorization of melanin by *Phanerochaete chrysosporium* NK-1

**Figure 1:**
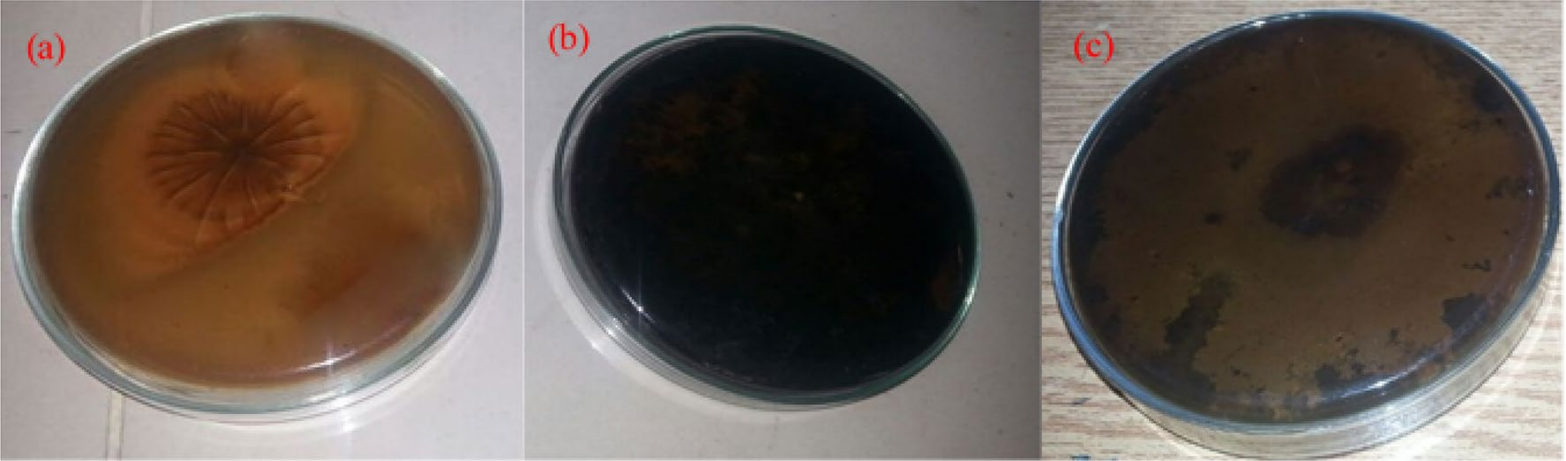
(**a**) Decolorization of melanin by the selected fungal strain: Decolorization of natural melanin by *P. chrysosporium* NK-1; (**b**) Control without treatment; (**c**) Decolorization of synthetic melanin by *P. chrysosporium* NK-1.

The fungal strains were tested for melanin decolorization. The fungi were spot-inoculated on the solid medium containing melanin and VA as the LiP inducer. After seven days of incubation *Phanerochaete chrysosporium* NK-1 showed the capability of decolorizing melanin, as shown in Fig. 1.

### Conditions for optimal melanin decolorization

#### pH

**Figure 2:**
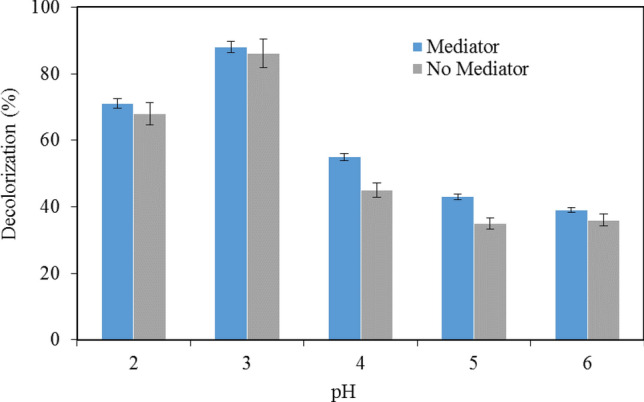
Effects of pH on the decolorization in the presence or in the absence of the mediator veratryl alcohol. Data points represent the means ± s.d., n = 3/group, *p*-value ≤ 0.05.

The purified enzyme was used for the decolorization of the synthetic melanin in vitro. The effects of different conditions on melanin decolorization were observed ^10^. The effects of pH on melanin decolorization by LiP were examined first. For this, melanin decolorization was performed at different pH conditions varying from 2.0 to 6.0 at a temperature of 30 °C both in the presence and absence of VA for an incubation period of 6 h. Results in Fig. 2 show that in general, decolorization of melanin in the presence of VA was higher in all pH conditions. Particularly, it is more prominent in higher pH conditions. VA can assist the formation of cation radicals ^11^. These radicals have much higher redox potential to attack C–C bonds non-specifically in melanin and result in decolorization ^12^. In the presence of VA, the highest melanin decolorization of 88% was achieved at pH 3 followed by 71% at pH 2, whereas the lowest melanin decolorization of 39% was observed at pH 6. In the absence of VA, the highest melanin decolorization of 86% was observed at pH 3 whereas the lowest melanin decolorization of 35% and 36% was observed at pH 5 and 6, respectively. Melanin decolorization by LiP in this study was highly efficient at lower pH conditions, i.e., 2 and 3 (Fig. 2), although other studies showed an optimal pH condition at 4 ^1^.

#### Temperature

**Figure 3:**
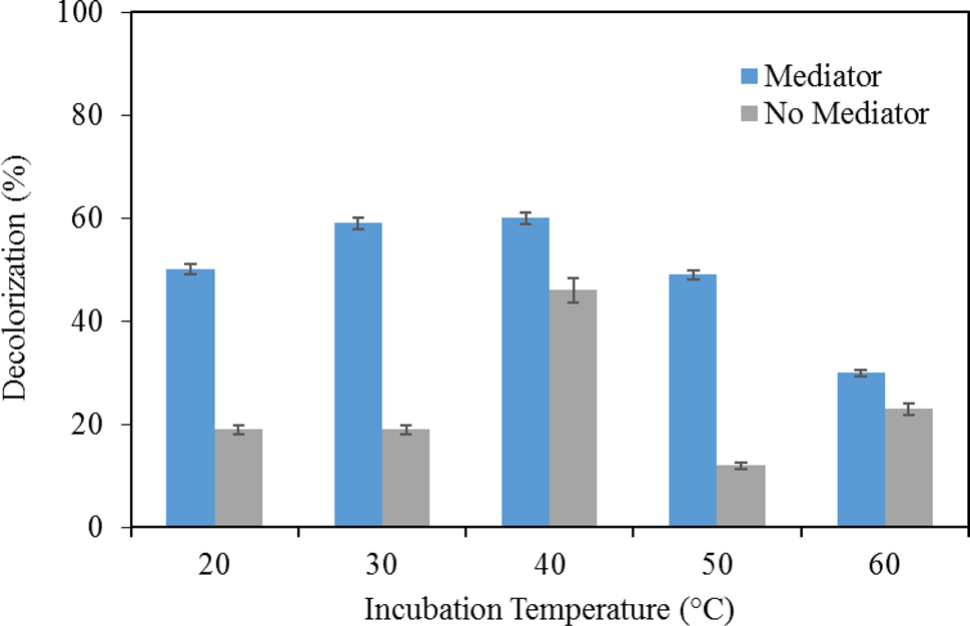
Effects of temperature on the decolorization in the presence or in the absence of the mediator veratryl alcohol. Data points represent the means ± s.d., n = 3/group, *p*-value ≤ 0.05.

Melanin decolorization by LiP was studied at different temperatures ranging from 20–60 °C both in the presence and absence of VA at an incubation period of 6 h. In the absence of VA, similar percentages (19%) of melanin decolorization was observed at temperature 20 and 30 °C (Fig. 3). The highest and lowest melanin decolorization in the absence of VA was observed at 40 and 50 °C, respectively. However, with VA in the solution, the melanin decolorization increased from 20 to 40 °C and decreased for up to 60 °C. In the presence of VA, the highest melanin decolorization is approximately 60% at 40 °C. The decolorization is generally lower because the experiment was conducted in a milder pH condition.

#### LiP concentration

**Figure 4:**
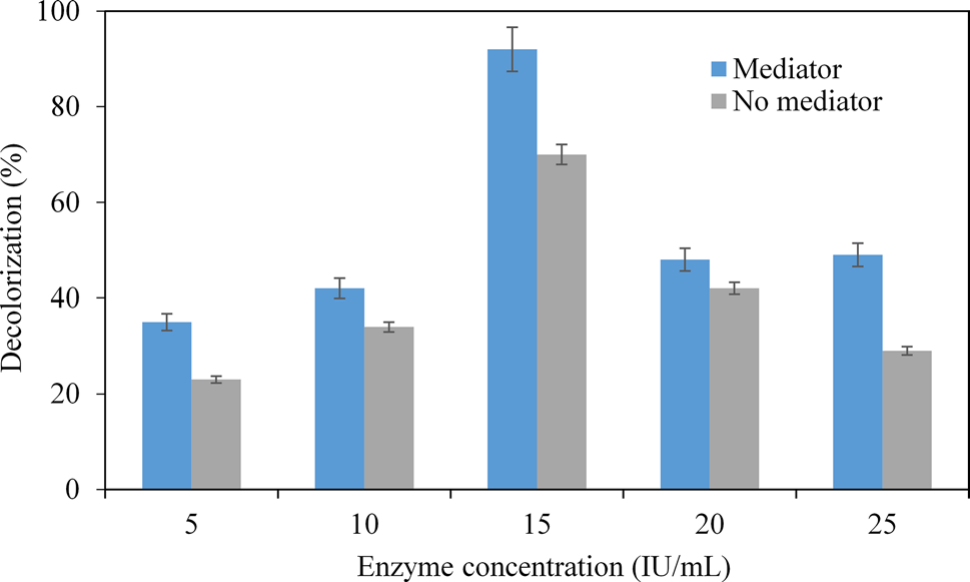
Effects of enzyme concentration on the decolorization in the presence or the absence of mediator veratryl alcohol. Data points represent the means ± s.d., n = 3/group, *p*-value ≤ 0.05.

The decolorization of melanin was also examined with variations of enzyme concentrations. Five different concentrations, i.e., 5, 10, 15, 20, and 25 IU/mL of LiP was examined for melanin decolorization in the presence and absence of VA, as shown in Fig. 4. Surprisingly, the most effective enzyme concentration was 15 IU/ml for both solutions with and without VA. After an incubation period of 6 h, the highest melanin decolorization was 92% in the presence of VA and 70% in the absence of the mediator using an enzyme concentration of 15 IU/mL, whereas the lowest melanin decolorization of 23% was observed in the absence of VA using 5 IU/mL of LiP. It is unclear that the decolorization is less effective with high enzyme concentrations, i.e., 20 and 25 IU/ml (Fig. 4).

#### Incubation time

**Figure 5:**
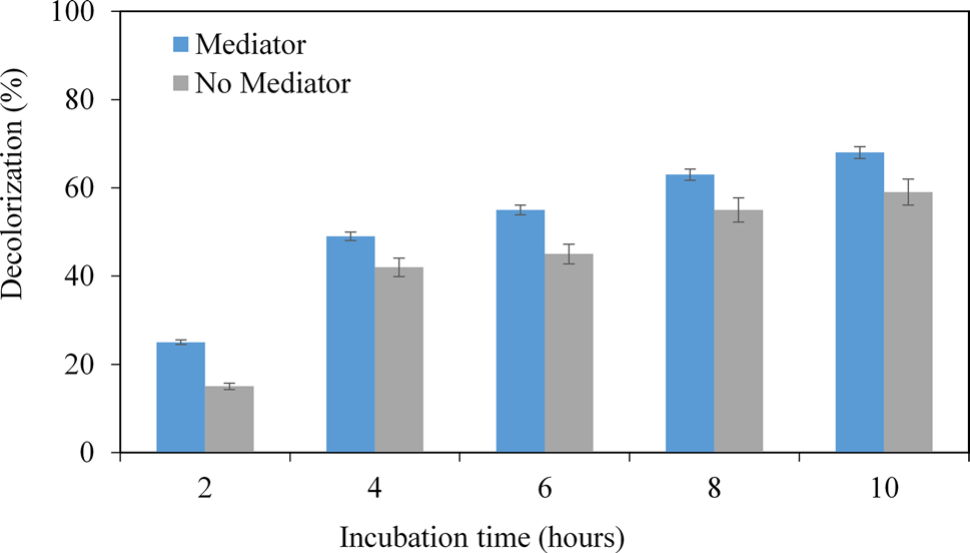
Effects of incubation time on the decolorization in the presence or in the absence of the mediator veratryl alcohol. Data points represent the means ± s.d., n = 3/group, *p*-value ≤ 0.05.

The decolorization experiments were carried out at 2, 4, 6, 8, and 10 h of incubation. These reactions were also carried out in the presence and absence of VA. The results showed that the decolorization efficacy is positively correlated to the incubation time, as expected (Fig. 5). The increase of delocalization extent was more prominent in the initial incubation. The highest melanin decolorization (68%) was observed after an incubation period of 10 h in the presence of VA and 59% in the absence of VA. The melanin decolorization was as low as 15% after 2 h of incubation without VA. Therefore, based on the test results, we have concluded that the optimal conditions are pH 3, 40 °C, 15 IU/ml, and 10 h incubation.

### SEM (Scanning electron microscope) and FTIR (Fourier-transform infrared spectroscopy) analyses

**Figure 6:**
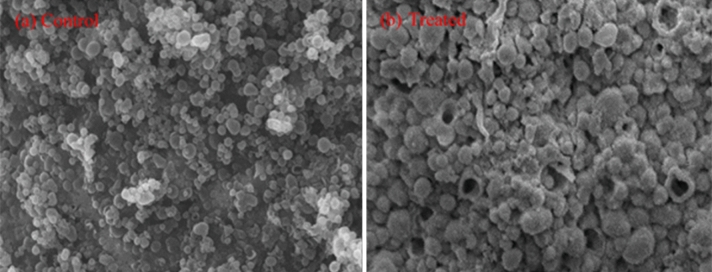
Surface morphology analysis of the control and treated samples by SEM: (**a**) Control standard melanin granules; (**b**) Treated melanin granules (showing void spaces and rough surfaces).

Morphological and structural changes in melanin were characterized by SEM and FTIR, respectively. The decolorized melanin was analyzed by SEM to observe any surface changes morphologically. Synthetic melanin without any enzyme treatment was used as the control. As shown in Fig. 6, there was void space on the treated melanin granules as compared to the untreated sample, suggesting melanin particles were attacked by the enzyme.

**Figure 7:**
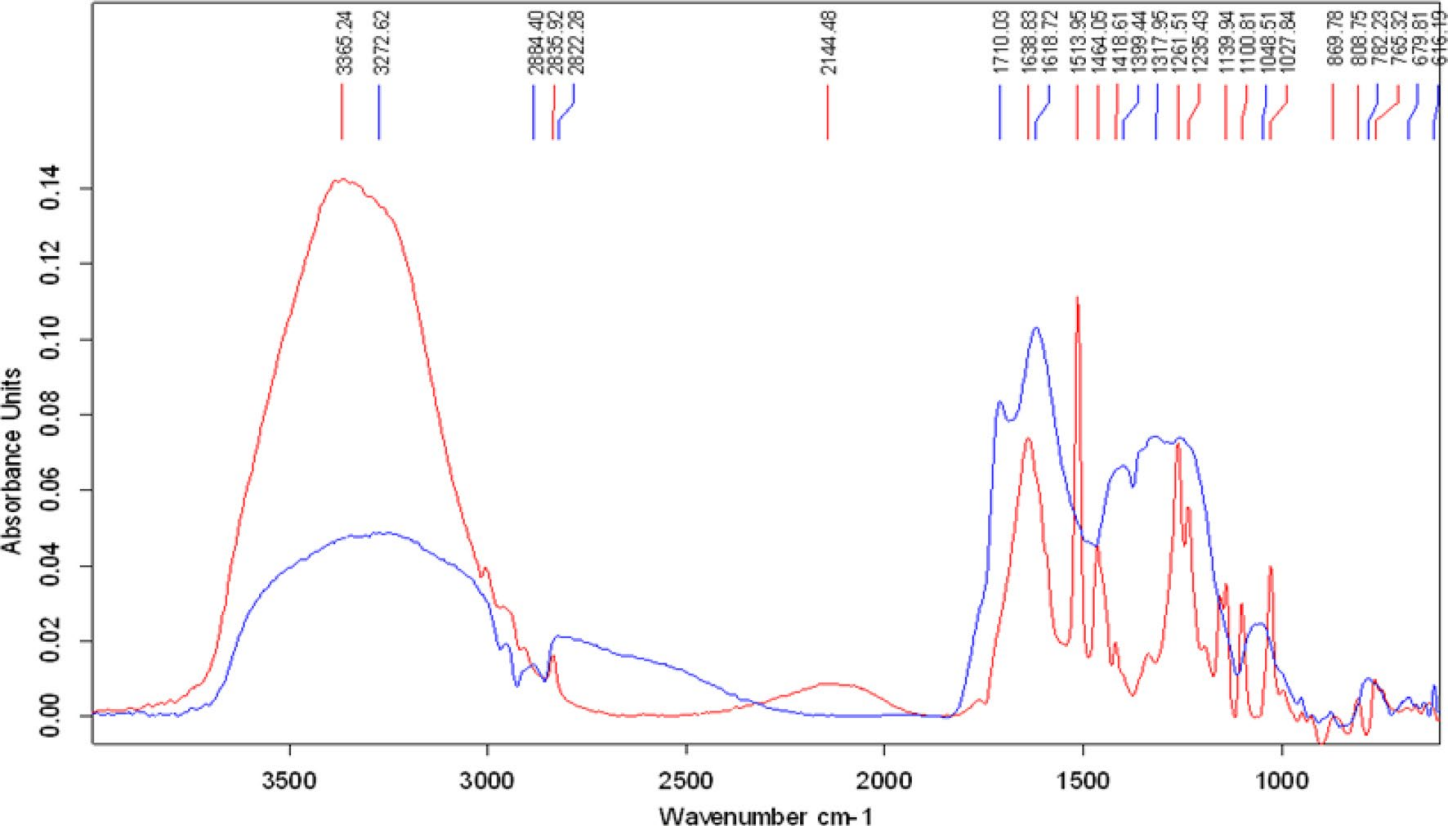
Structural changes of melanin by FTIR analysis (blue: control; red: treated).

Decolorized melanin was also analyzed by FTIR with the untreated melanin as the control (Fig. 7). The molecular fingerprints of both decolorized and untreated melanin were obtained and compared to confirm any structural changes in the treated melanin ^13^. FTIR spectra of the melanin (C_18_H_10_N_2_O_4_) showed a broad absorption spectrum at 3272 cm^−1^ which could be attributed to the characteristic O–H stretching or N–H stretching vibrations of the carboxylic acid, and phenolic groups. The changes in the FTIR spectrum of the treated melanin were noticeable. A broadband at wavenumbers 3000–3500 cm^−1^ indicates the presence of -NH and -OH groups in both control and treated samples. However, the intensity of the treated sample is significantly higher than the control sample, suggesting the treatment had additions of these functional groups to the structure by enzymatic attacks. The peaks at 2884.4 cm^−1^ and 2822 cm^−1^ indicated the presence of alkyl groups in the control while in the decolorized melanin, the peak at wavenumber 2884.4 cm^−1^ was absent. The peak at 2822 cm^−1^ deviated to wavelength 2835.92 cm^−1^ which represented the structural changes in the treated melanin in this region as well. The peak at the wavenumber 1710 cm^−1^ in the control was attributed to the presence of carboxylic acid. This peak was also absent or reduced to a large extent in decolorized melanin, again confirming the structural changes in the treated melanin. There were also some changes in the fingerprint region. Between wavenumbers 1500–500 cm^−1^, for example, the presence of new peaks in the treated melanin at 1513, 1464, and 1139 cm^−1^ represented structural changes. A new peak at 2144 cm^−1^ was also detected in the decolorized melanin.

### Cytotoxicity effects

Cytotoxicity of the degraded melanin and LiP was investigated using the brine shrimp cytotoxicity method ^14,15^. In the present study, cytotoxic effects of the treated melanin and LiP were low with shrimp larvae viable rates of ≥ 90% (Table 1). The increase of LiP concentration had no impact on the viability of the shrimp larvae for up to 80 μl/mL (~ 80 IU/ml), suggesting its low cytotoxic activity. In the positive controls with VA, the mortality rate was 100%, suggesting that cautions should be taken during the application of LiP in the presence of VA as a mediator in the medical and cosmetic fields.

**Table 1:** Cytotoxic effect of treated melanin and lignin peroxidase.

Sample	Conc. (µL/mL)	Nauplii used	Nauplii alive	Nauplii died	Mortality (%)
Negative control	NA	10	10	0	0
Positive control (VA)	50	10	0	10	100
Positive control (TE buffer)	50	10	8	2	20
Positive control (melanin)	50	10	8	2	20
Cytotoxic effect of treated melanin	20	10	10	0	0
	40	10	9	1	10
	60	10	9	1	10
	80	10	10	0	0
	100	10	10	0	0
Cytotoxic effect of lignin peroxidase	20	10	10	0	0
	40	10	10	0	0
	60	10	10	0	0
	80	10	10	0	0
	100	10	9	1	10

## Discussion

The toxicity of chemical additives used in skin whitening formulations has raised serious concerns and demands alternative approaches offering health and safety benefits. In the present study, an enzymatic approach was evaluated with the possibility to replace harsh chemicals with eco-friendly natural products. The use of enzymes as an active ingredient of skin whitening formulations can be very advantageous due to their high substrate specificity and no cytotoxic effects ^16^. An indigenous isolate of *Phanerochaete chrysosporium* was used to produce LiP under submerged fermentation conditions. Previously, *P. chrysosporium* has been reported to secrete extracellular enzymes, capable of degrading melanin ^17^. The process for the optimal production of LiP was studied using Placket–Burman design. Besides the optimal level of medium components (supplementary data), pH and temperature play an important role both in terms of enzyme production and its catalytic activity. The optimal pH for the maximum production of LiP from *P. chrysosporium* was found to be acidic (pH 3). It has been reported that during the growth of the fungus, it secretes organic acids leading to a pH drop to as low as 2 which promotes pH-induced acid-catalyzed hydrolysis of complex biopolymers. In addition, *P. chrysosporium* produces acid-stable LiP which causes melanin decolorization. The possible reason for improved catalysis of melanin by LiP could be attributed to the role of supramolecular interactions that help maintain appropriate protein conformation at higher proton concentrations ^1^.

**Figure 8:**
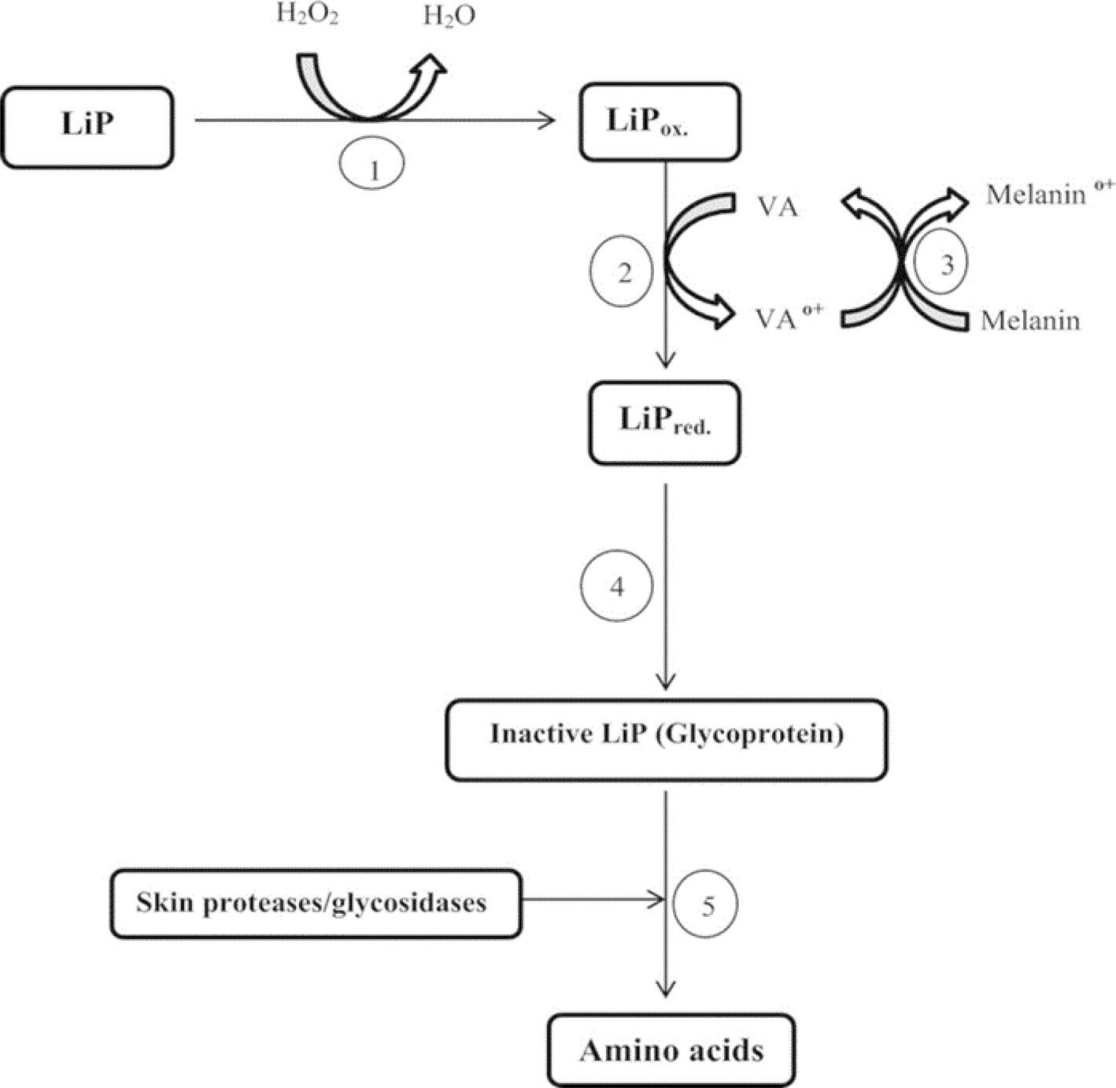
Proposed mechanism of the melanin decolonization by LiP.

In the case of temperature, the highest fungal growth and enzyme production was achieved at 40 °C, which corresponds to the natural habitat of *P. chrysosporium.* The ability of LiP to degrade melanin is based on the structural similarity between melanin and lignin ^18^. The efficacy of the degradation of melanin by LiP in vitro has been correlated to a number of factors such as pH, temperature, enzyme concentration, and incubation time as well as the existence of VA. LiP has the ability to oxidize VA to its cation radicals (VA∙^+^) and serve as a redox mediator that further decolorize melanin, as shown in Fig. 8.

H_2_O_2_ is an essential substrate as a final electron acceptor during the melanin decolorization. LiP is easily inactivated in their excess concentration which induces the rupture of the heme structure or formation of the inactive compound III. It is reported that this inhibitory effect can be subdued by oxidation of VA to VA cation radical ^1^. LiP oxidizes VA to VA cation radicals, which acts as a redox mediator to decolorize melanin. VA is a natural compound produced from white-rot fungi which is expected to have fewer side effects. However, the presence of the mediator VA has resulted in high mortality of the tested organisms, compared to the low cytotoxic effects of treated melanin and LiP. It should be carefully tested in the formulation of health and skincare products.

We found 88% decolonization of lignin at pH 3 which indicated that low pH favors direct contact of the enzyme with VA. Similar findings were also reported by Sung et al. (2019), where they found that low pH must be maintained for optimal catalytic efficiency. The decolorization increases slowly within the lower end of the tested temperature (20–40 °C) and decreases rapidly as the incubation temperature continues to increase, suggesting denaturation may occur at higher temperatures. It is generally expected that the degradation rate of melanin increases with increasing enzyme concentrations. Surprisingly, our results showed a decrease in decolorization efficacy with increasing enzyme concentrations. At high concentrations, the low activity might be attributed to other reaction conditions, such as temperature and pH. The changes int these conditions could cause changes in supramolecular architecture of the protein to compromise the enzyme–substrate binding efficiency and lead to a decreased reaction rate ^1^. However, this particular aspect needs further investigation. On the contrary, the degradation of melanin increases with the increment of incubation time suggesting the enzyme was stable during the treatment period. In all the experimental conditions, decolorization was found more effective in the presence of VA because it facilitates formations of radicals with high redox potential that could un-specifically attack carbon–carbon bonds. The SEM and FTIR analyses also confirm that the enzymatic treatment has rendered morphological and structural changes of the melanin.

Based on our results, it is suggested that *P. chrysosporium* can be used as a potent biological resource for the commercial production of LiP for its applications in the medical and cosmetic industries particularly for the development of new cosmetic whitening agents.

## Materials and methods

### Isolation and identification of fungal strains

The fungal isolation was done from a contaminated site used as a dumped site for wood materials for a long time. The sample was taken in sterile bottles and transferred to the lab for purification. Purification was done on a seaboard dextrose agar medium. Four different fungal colonies were isolated and purified from the sample. Screening for the best strain was done based on the melanin decolorization and biomass production in the presence of melanin as the only carbon source in the mineral salt medium (MSM). Based on the screening, the selected organism was identified by sequencing the DNA of 5.8S rRNA and 18S rRNA using universal primers ITS-1 and ITS-4 ^10^. Their Genomic DNA was extracted according to the method of Anderson et al. ^19^. Following purification, the sequencing of the PCR products was performed. The sequences were aligned using BLAST tool at the NCBI and homologs were analyzed for phylogeny using Molecular Evolutionary Genetic Analysis (MEGA). On the basis of a maximum likelihood, a neighbor-joining tree was constructed for the identification of the isolated fungal strain. The sequences were submitted to the NCBI GenBank to with an accession number of KX064682.1. Phylogenetic analysis revealed that the isolate KX064682.1 was a strain of *Phanerochaete chrysosporium* NK-1.

### Plate assay for melanin decolorization

The white-rot fungus, *Phanerochaete chrysosporium* NK-1, was used for melanin decolorization. *Phanerochaete chrysosporium* is known to produce LiP. Synthetic melanin (B049-97–6) was used as the model melanin in all the melanin decolorization experiments. A modified medium with VA ^2^ as the LiP inducer was used to test the ability of melanin decolorization of the fungus. Petri dishes were used to culture *Phanerochaete chrysosporium* NK-1. The medium was prepared by dissolving 10 g of glucose, 2 g of malt extract, 4 g of MgSO_4_·7H_2_O, 1 g of KH_2_PO_4_, 0.01 g of FeSO_4_, 0.005 g of ZnSO_4_, 0.2 g of synthetic melanin, and 15 g of agar in 1-L distilled water. Inoculated Petri plates were incubated at room temperature in darkness. The extent to which melanin decolorized beneath and around the colony of the fungus was observed. It was found that the fungus is capable of decolorizing melanin and further used to prepare enzymatic solutions.

### Preparation of enzyme for melanin decolorization

The liquid medium of the culture contained 10 g of glucose, 0.2 g of yeast extract, 0.07 g of VA, 3.0 g of tartaric acid, 1 g of tween 80, 0.2 g of KH_2_PO_4_, 0.146 g of CaCl_2_·2H_2_O, 0.157 g of K_2_HPO_4_, 0.05 g of MgSO_4_·7H_2_O, 42.5 mg of ZnSO_4_·7H_2_O, 7.0 mg of CoCl_2_·6H_2_O, 7.0 mg of CuCl_2_·2H_2_O, 0.54 mg of FeCl_3_, 0.9 mg of NaCl and 0.2 mg of synthetic melanin per liter of distilled water ^20^. The pH of the medium was adjusted to 4.0 with NaOH and HCl solutions. 250 ml flasks containing aliquots of 100 ml culture medium were autoclaved and inoculated with a 5 ml inoculum. The flasks were then incubated on a rotatory shaker at 30 °C for 15 days. Duplicate samples were collected for LiP activity assays at an interval of 24 h.

### Enzyme assay

Reagent for LiP assay included 250 mM sodium tartrate buffer pH 5.5, 10 mM VA, and 4 mM H_2_O_2_ (30%). The enzyme assay was carried out in silica cuvette, and the absorbance was measured at 310 nm by a UV–visible spectrophotometer (Shimadzu, Kyoto, Japan). Absorbance was monitored at time 0–30 s under ambient conditions. The activity of the enzyme was calculated according to Beer’s Law, Eq. (1) ^21^.

1 $$ c = A/\varepsilon d $$

where c, the concentration of the attenuation species; A, the optical absorptivity; ε, the molar attenuation coefficient; d, the length of the optical path. Measuring enzyme activity involves the determination of the change in concentration over time, Eq. (2).

2 $$ \frac{\Delta c}{{\Delta t}} = \left( {\Delta As - \Delta Ab} \right)/\varepsilon \cdot \Delta t $$

### Optimization for enhanced production of LiP and decolorization of melanin in vitro

Optimization for the enhanced production of LiP from *Phanerochaete chrysosporium* NK-1 using melanin as the substrate was carried out using a Placket Burman design. A full-length Placket was based on the fact that it undermines interactive effects between different variables whereas it considers only linear trends, Eq. (3) ^22^.

3 $$ Y = \beta o + \sum \beta ixi \left( {i = 1, \ldots ,k} \right) $$

where Y represents the response and β symbols indicate the regression coefficient and k is the number of studied factors. A total of 9 factors were studied to achieve the highest concentration of LiP from *Phanerochaete chrysosporium* NK-1 for the decolorization of melanin present in the medium. The Placket–Burman design was constructed with 12 total experimental runs, as shown in Table 2. Experiments were conducted for a total of 35 days and the response was measured (LiP activity IU/mL). The experimental data were analyzed using the statistical software ‘Design Expert 9’. The optimized medium was used for the enhanced production of LiP and purified by ammonium precipitation.

**Table 2:** Optimization of LiP production using Placket–Burman design.

Run Numbe	Time (days)	Temperature (°C)	Glucose (g/l)	Fructose (g/l)	Yeast Extract (g/l)	Peptone (g/l)	Inoculum Size (ml)	Substrate	Mediator (ml)	LiP (IU/ml)
1	35	40	10	20	5	5	2	10	0.1	383
2	35	20	20	20	5	2	2	10	0.01	418
3	35	40	20	10	2	2	5	10	0.1	576
4	18	20	10	20	2	5	5	10	0.1	221
5	18	20	10	10	2	2	2	10	0.01	171
6	18	40	20	10	5	5	5	10	0.01	401
7	35	40	10	10	2	5	2	100	0.01	368
8	35	20	20	20	2	5	5	100	0.01	489
9	18	40	10	20	5	2	5	100	0.01	300
10	18	40	20	20	2	2	2	100	0.1	547
11	35	20	10	10	5	2	5	100	0.1	402
12	18	20	20	10	5	5	2	100	0.1	475

### Optimization of enzyme production and purification

A submerged medium was used to determine the optimal incubation time in terms of LiP activities. As shown in Supplemented Fig. 1, the culture solution has a maximal enzymatic activity on the 8th day, being ~ 140 IU/ml. Therefore, the incubation time of 8 days was used for producing the enzyme solution.

A total of 12 experiment runs were conducted to determine the factors (9) that have impacts on LiP activities. Among the tests, the response values of the LiP activity range from 171 IU/ml to 576 IU/ml, as shown in Supplemented Fig. 2. The run with the highest LiP activity was run 3 (576 IU/ml) followed by run 10 (547 IU/ml). According to regression analysis, temperature, time, glucose concentration, substrate concentration, and the mediator have positive effects on LiP activity. The effect of yeast extract was found to be negligible. Other factors have negative effects. The optimal conditions for the LiP production are time: 35 days, temperature: 40 °C, glucose (per liter): 20 g, fructose: 10 g, yeast extract: 2 g, peptone: 2 g, inoculum: 10 ml, substrate mediator: 0.1 ml. These conditions were then used for the enhanced production of LiP which was purified by ammonium precipitation and gel chromatography (Sephadex G-75) methods.

Enzyme extraction at optimized pH, temperature, and incubation time was conducted. Sephadex G75 gel filtration column was used to separate the fraction of the protein content from the cell lysate with respect to the density gradient according to the manufacturer’s instruction. The abundance of proteins in the crude extract was also indicated through the number of bands that appeared on a sodium dodecyl polyacrylamide gel (SDS-PAGE) analysis. The molecular weight of the purified enzyme was 46.0 kDa as calculated from the SDS-PAGE analysis (Supplemented Fig. 3). The enzymatic activities after ammonium precipitation and purification by gel chromatography are 684.0 and 957.6 IU/ml, respectively.

The purified enzyme was used for the decolorization of melanin at different physiochemical conditions, including pH, temperature, incubation time, and enzyme concentration. Synthetic melanin (B049-97-6) was used as the model melanin in all the melanin decolorization experiments according to ^23^.

### Decolorization experiments

#### Effects of pH on melanin decolorization

To observe the effects of pH on decolorization of melanin by LiP, melanin decolorization reactions were carried out at pH extending from 2.0–6.0. The reaction mixture contained 10 µl of the crude enzyme dissolved in a TE buffer and 1990 µl of 0.02% w/v of melanin. These reactions were performed both in the presence and absence of VA. The reaction mixtures were incubated at a temperature of 30 °C for 6 h. Control experiments were run in parallel by replacing active LiP with the denatured enzyme that was boiled at 95 °C. The reaction mixtures were monitored for melanin decolorization using a spectrophotometer at a wavelength of 540 nm before and after incubation. The decolorization efficiency of the enzyme was expressed in percent (%). Melanin decolorization was calculated by Eq. (4) ^11^.

4 $$ DC\% = \frac{{A_{0} - A_{f} }}{{A_{0} }}*100 $$

where DC% representing a percentage of decolorization; A_0_ and A_f_ representing absorbance before and after enzyme treatments.

#### Effects of temperature on melanin decolorization

The melanin decolorization reactions were carried out at temperatures extending from 20–60 °C. The reaction mixture contained 10 µl of the crude enzyme dissolved in a TE buffer and 1990 µl of 0.02% w/v of melanin at pH 4 both in the presence and absence of VA. The reaction mixtures were incubated for a period of 6 h and the percentage of melanin decolorization was obtained.

#### Effects of incubation time on melanin decolorization

For optimization of incubation time, melanin decolorization reactions were carried out at incubation times extending from 2–10 h. The reaction mixture contained 10 µl of the crude enzyme dissolved in a TE buffer and 1990 µl of 0.02% w/v of melanin at pH 4 in the presence and absence of VA. The reaction mixtures were then incubated at a temperature of 30 °C and the percentage of melanin decolorization was obtained.

#### Effects of enzyme concentration on melanin decolorization

To optimize the enzyme concentration, melanin decolorization reactions were carried out at different enzyme concentrations extending from 5–25 IU/ml in the reaction mixtures. 2 ml of the reaction mixtures containing 5, 10, 15, 20, and 25 IU/ml of the crude enzyme and 0.02% w/v of melanin were prepared both in the presence and absence of VA. The reaction mixtures were then incubated at a temperature of 30 °C for 6 h and the melanin decolorization was measured.

### SEM analysis of degraded melanin

Surface Morphological changes of the melanin were analyzed by a scanning electron microscopy (JEOL JSM-5910, Peabody, MA, USA) ^24^. Untreated melanin was used as the control. The samples were dried and fixed on copper stubs (10 × 10 mm^2^) with double-sided carbon tape (sticky on both sides). The stubs were washed with detergent and dried with a heat gun drier (KADA 85 U/SMD) at 200 °C for 2 min prior to sample fixation. Silver paste conduction (SPI-CHEM, West Chester, PA, USA) was used to ensure the electron beam conduction. Gold was deposited onto the sample with plasma created with high voltage (25 mA current for 50 s) and vacuum (10^−2^ ATM) conditions. The surface morphology of the samples was examined at 3000 magnification power.

### FTIR analysis of decolorized melanin

The reactions or samples that showed the highest decolorization of melanin in the optimization experiment were further analyzed by FTIR (Model No 56, Merlin, Germany) spectroscopy. The samples were centrifuged (10,000 rpm for 1 min) and air-dried as a preparation step for the FTIR analysis ^20^. A qualitative analysis of melanin samples was performed using UV–Vis near infrared (NIR) spectrometer Lambda 900/Perkin Elmer Instruments. The spectra were recorded in the range of 1000 to 3500 cm^−1^. Samples were prepared in KBr pellets with powder dispersion. The untreated synthetic melanin was used as the control in this analysis.

### Cytotoxicity of degraded melanin and LiP

Brine shrimp lethality tests were conducted to check the cytotoxicity of the degraded melanin ^14,15^. The eggs of the brine shrimp were hatched in a rectangular dish having artificial seawater. The dish contained a plastic divider with small holes and divided the dish into two unequal compartments. The eggs were speckled in the larger compartment and covered to avoid any light penetration, whereas the smaller compartment was illuminated with a lamp on top of it. The hatched larvae were attracted by the light towards the small compartment. After 48 h of incubation, the mature nauplii were collected from the lit compartment using a pasteurized pipette.

Five different dilutions (20, 40, 60, 80, and 100 µL/ml) of the degraded melanin and LiP were tested for cytotoxicity effects. The degraded melanin was added to the small test tubes each having 2 mL of seawater with 10 live brine shrimp larvae. The negative control was the solution having artificial seawater with the brine shrimp larvae. Three positive controls were prepared to contain untreated melanin in the artificial seawater with larvae.

### Statistical analysis

The experimental data were analyzed using the statistical software ‘Design Expert 9’. Graphical images were drawn by using means of three experimental trials with calculated standard deviation. Statistical significance of the experimental run was calculated by ANOVA.

## Supplementary information

Supplementary Information.

## Data Availability

The authors confirm that the data supporting the findings of this study are available within the article [and/or] its supplementary materials.
