# Supplementary material for: Enzymatic decolorization of melanin by lignin peroxidase from Phanerochaete chrysosporium
Source: Sci Rep. 2020 Nov 19;10:20240. doi: 10.1038/s41598-020-76376-9 (PMC7677534; doi:10.1038/s41598-020-76376-9)
Supplement: Supplementary file 1 — Supplementary Information. [file 41598_2020_76376_MOESM1_ESM.docx]

**Enzymatic decolorization of melanin by lignin peroxidase from *Phanerochaete chrysosporium***

Beenish Sadaqat^1^, Nazia Khatoon^2^, Aneela Younas Malik^1^, Asif Jamal^1^, Uzma Farooq^1^, Muhammad Ishtiaq Ali^1*^, Huan He^3^, Fang-Jing, Liu^3^, Hongguang Guo^4^, Michael Urynowicz^5^, Qiurong Wang^6^, Zaixing Huang^3,5*^

^1^Department of Microbiology, Quaid-i-Azam University, 45320, Islamabad, Pakistan

^2^Instiute of Space Technology, 44000, Islamabad, Pakistan

^3^Key Laboratory of Coal Processing and Efficient Utilization of Ministry of Education, School of Chemical Engineering and Technology, China University of Mining and Technology, Xuzhou, 221116, China

^4^College of Safety and Emergency Management and Engineering, Taiyuan University of Technology, Taiyuan 030024, China

^5^Department of Civil and Architectural Engineering, University of Wyoming, 82071, USA

^6^Department of Animal Science, University of Wyoming, Laramie, WY 82071, USA


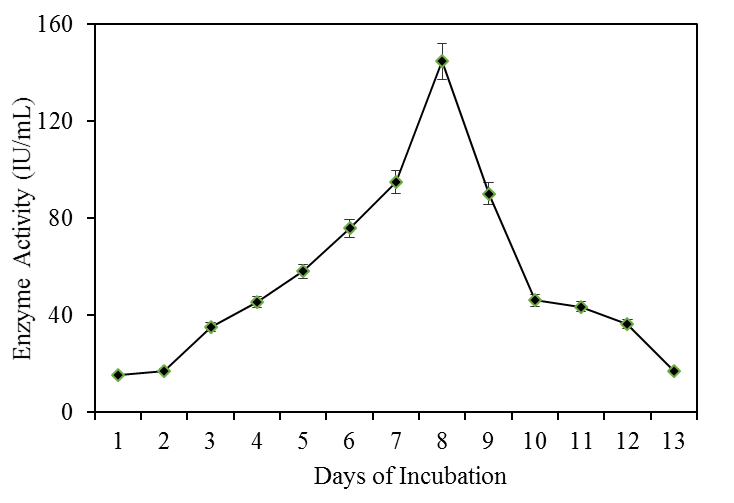


Supplementary Figure 1 Enzyme activity over incubation time. Data points represent the means ± s.d., n=2/group.


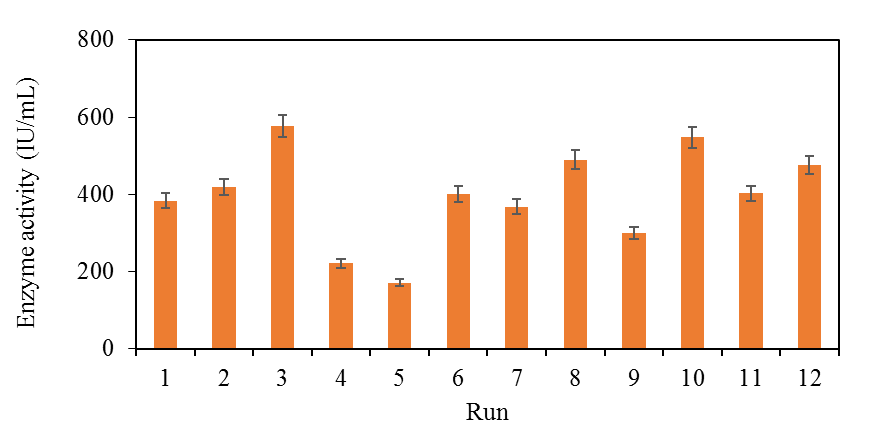
Supplementary Figure 2 Optimization of enzymatic activity under different combinations of 9 factors in 12 experimental runs. Data points represent the means ± s.d., n=3/group.


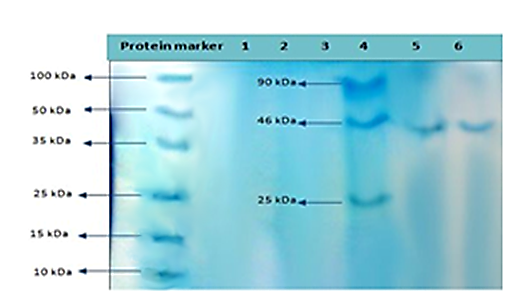
Supplementary Figure 3 Molecular weight identification of the purified fraction from the gel filtration chromatography by separation of characteristic band on SDS acrylamide gel.
